# Circulating Interleukin-6 and CD16 positive monocytes increase following angioplasty of an arteriovenous fistula

**DOI:** 10.1038/s41598-022-05062-9

**Published:** 2022-01-26

**Authors:** Seran Hakki, Emily J. Robinson, Michael G. Robson

**Affiliations:** 1 School of Immunology and Microbial Sciences, Faculty of Life Sciences and Medicine, King’s College London, Guy’s Hospital, London, SE1 9RT UK; 2 School of Population Health and Environmental Sciences, Faculty of Life Sciences and Medicine, King’s College London, London, UK

**Keywords:** Immunology, Nephrology

## Abstract

Arteriovenous fistulas are the ideal form of vascular access that allows provision of haemodialysis. Stenotic lesions caused by neointimal hyperplasia commonly occur resulting in patients requiring a fistuloplasty. This is effective but there is a high recurrence rate. We sought to investigate the effects of a fistuloplasty on monocyte populations. Blood samples were taken from patients before and after their fistuloplasty procedure. Samples were analysed using flow cytometry, ELISA and Luminex assays. Univariate cox regression was carried out to investigate associations with post fistuloplasty patency. At 1–2 days post fistuloplasty, the proportion of classical (CD14++CD16−) monocytes decreased (*p* < 0.001), whilst intermediate (CD14++CD16+) and non-classical (CD14+CD16+) monocytes increased (both *p* < 0.01) in a cohort of 20 patients. A time course study carried out in 5 patients showed that this was due to an increase in absolute numbers of non-classical and intermediate monocytes. Higher levels of non-classical monocytes pre-fistuloplasty were associated with an increased risk for patency loss (*p* < 0.05). We measured 41 soluble factors in plasma samples taken before a fistuloplasty in 54 patients, with paired post-fistuloplasty samples (1–2 days) available in 30 patients. After correcting for false discovery, the only factor with a significant change in level was IL-6 (*P* = 0.0003, q = 0.0124). In a further time-course study in 6 patients, peak level of IL-6 occurred 2–3 h post fistuloplasty. This study demonstrates that there is a systemic inflammatory response to the fistuloplasty procedure and that monocyte subsets and IL-6 may be important in the pathophysiology of restenosis.

## Introduction

Haemodialysis is the most commonly used form of long-term renal replacement therapy and one of the main determinants of the effectiveness of a patient’s haemodialysis is the patency their vascular access. A native arteriovenous fistula (AVF) is the ideal form of vascular access for long term haemodialysis in most patients because of its overall superior patency and mortality rate^1^. The increased mortality associated with central venous catheters and arteriovenous grafts is primarily due to infection^1^.

Stenotic lesions, which are structures resulting in an abnormal narrowing of the blood vessel lumen, thus limiting blood flow, are a common cause of AVF failure. The initial treatment is usually a radiological fistuloplasty. Despite the initial success of the procedure, the benefits may be short-lived as restenosis is frequent. Retrospective studies have reported post-intervention primary patency rates of around 60–70% at 6 months and 40–50% at one year^2–8^. Similar patency rates have been reported in recent prospective randomised controlled trials^9–11^.

The stenotic lesions of the fistula are caused by neointima hyperplasia (NIH). NIH refers to the proliferation and migration of vascular alpha smooth muscle actin expressing cells into the vessel’s intima layer. Monocytes are likely to be important in the pathophysiology of NIH as two animal models have demonstrated that the depletion of monocytes with clodronate prevented NIH formation^12,13^. Furthermore, one of the few laboratory studies looking for biomarkers for restenosis following a fistuloplasty procedure found that monocyte chemoattractant protein (MCP-1) is elevated up to two weeks post fistuloplasty and an early increase in MCP-1 at two days post procedure was an independent predictor of restenosis^14^. Therefore, previous data suggested that monocytes may play a role in the processes leading to restenosis following a fistuloplasty.

Circulating monocytes can be subdivided into classical (CD14++CD16−) intermediate (CD14++CD16+) and non-classical (CD14+CD16+) monocytes. These subsets are phenotypically and functionally different. Classical monocytes are the most prevalent subset and considered important for microbial defence, whilst CD16+ monocytes crawl on the endothelium, rending them important for innate surveillance and repair of tissues^15^. The aim of this study was to assess changes in monocyte subsets and soluble factors following a fistuloplasty procedure, and to explore for potential associations between these factors and outcome.

## Results

### Patient demographic features

**Figure 1:**
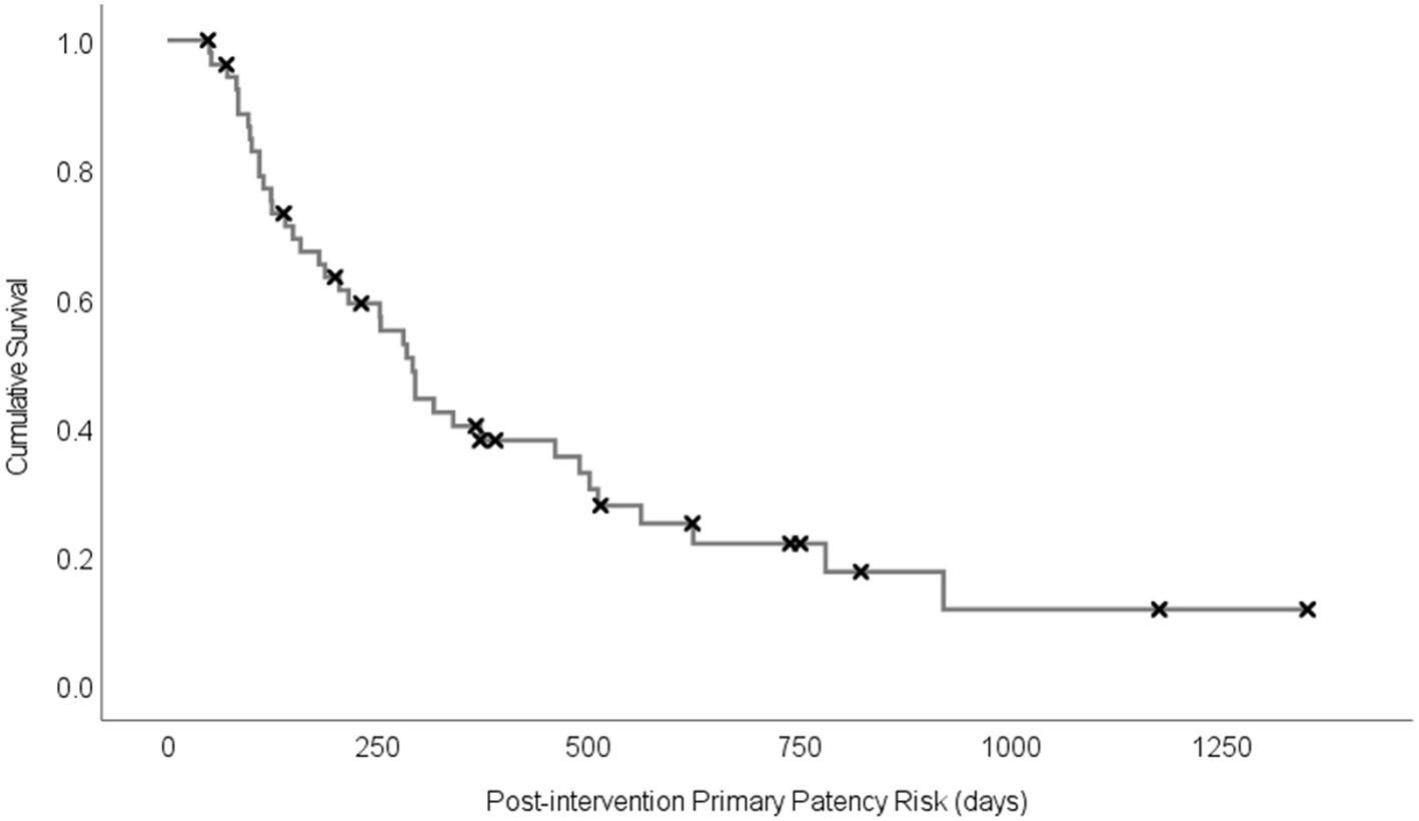
Kaplan–Meier plot of days to loss of post-intervention primary patency; X’s indicate patient censoring.

A total of 54 patients who required a fistuloplasty procedure were included in this study. A summary of the demographic features of this population are shown in Table 1 and are typical for the local dialysis population. Patients were followed up for a minimum of one year or until censored. During this period, 40 patients reached the end of post-intervention primary patency (PIPP) after a median time of 204 days. Figure 1 shows a Kaplan–Meier survival curve for the entire cohort. Three patients were censored in the first six months before reaching the endpoint, and of the remaining 51 patients, 18 (35%) lost PIPP by six months. Paired pre and post fistuloplasty blood samples could only be obtained from 30 of these 54 patients. Table S1 details each individual participant’s demographic and clinical features, in addition to indicating the experiments in which their samples were used. The patients all had a clinical indication for the fistuloplasty, with the major reason for each given in Table S1. The use of antiplatelets, oral anticoagulants, statins, and immunosuppressive medication is also given in Table S1. Eight of the fistulas had not been used for dialysis at the time of intervention and this is also indicated.

**Table 1:** Table summarising the demographic information of the 54 patients within this study.

	n (%) unless stated
Mean patient age (years ± SD)	60 ± 14
Female	23 (42)
Male	31 (58)
Asian	8 (15)
White	19 (35)
Black	27 (50)
Basilic vein transposition	15 (28)
Brachiocephalic	25 (46)
Radiocephalic	14 (26)
Age of fistula (Median years ± IQR)	1.46 ± 3.25
Previous radiological interventions	13 (24)
Previous thrombosis	2 (4)

### Changes in monocyte subsets following a fistuloplasty procedure

**Figure 2:**
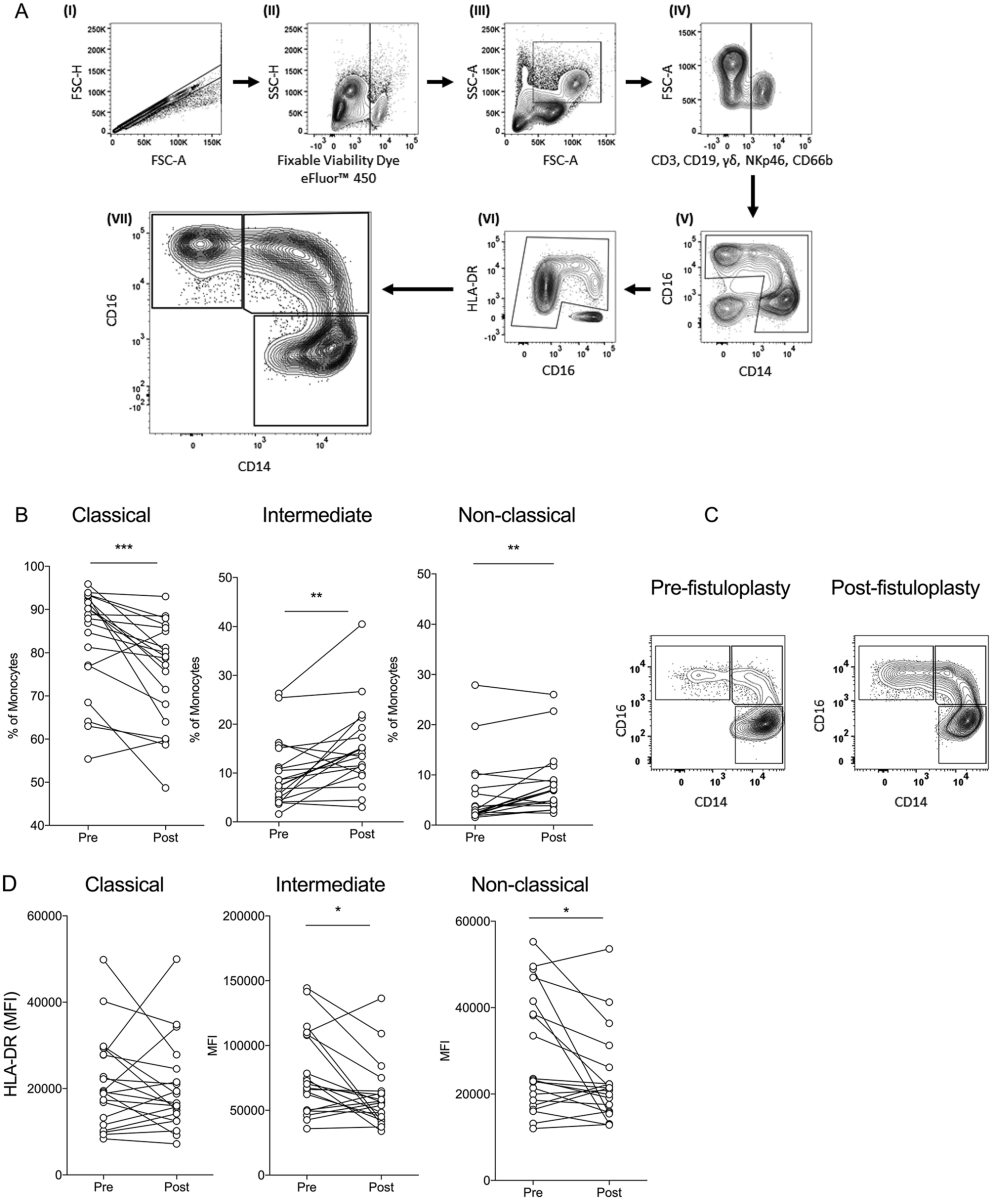
Changes in monocyte subsets 1–2 days post fistuloplasty. (**A**) The gating strategy for monocyte subsets from cryopreserved PBMCs. Singlets (I) were gated for viability (II) and then gated as potential monocytes by drawing a generous gate based on forward and side scatter properties (III). CD3+, CD19+, γδ+, CD66b+ and NKp46+ cells were dumped (IV). The remaining cells were then gated based on monocyte’s characteristic morphology when gated for CD14 and CD16 (V). Contaminating cells were further removed by gating HLA-DR with CD16 (VI). Monocyte subsets were then gated based on CD14 and CD16 expression (VI). Image created with Flowjo version 10.6.1 (www.flowjo.com) (**B**) The percentages of classical, intermediate and non-classical monocytes pre and 1 day post fistuloplasty. n = 20 paired pre and post fistuloplasty samples. (**C**) Representative plot of monocyte subsets pre and 2 days post fistuloplasty from a patient. (**D**) Median fluorescence intensity levels of HLA-DR in classical, intermediate and non-classical monocytes pre and 1 day post fistuloplasty. n = 20 paired pre and post fistuloplasty samples. *p < 0.05, **p < 0.01, ***p < 0.001.

First, we sought to establish whether there were any changes in the major monocyte populations in the peripheral blood following a fistuloplasty procedure. We tested paired cryopreserved peripheral blood mononucleated cells (PBMCs) from 20 patients before and 1–2 days after their fistuloplasty procedure using flow cytometry. The gating strategy used is shown in Fig. 2A. We found that there was a significant shift in the monocyte populations towards a CD16 expressing phenotype post fistuloplasty as the percentage of classical (CD14++CD16−) monocytes significantly decreased (*p* < 0.001), whilst the percentage of intermediate (CD14++CD16+) and non-classical (CD14+CD16++) monocytes significantly increased (*p* < 0.01) (Fig. 2B). Figure 2C shows a representative flow cytometry plot of this shift. We also observed that there was a small yet statistically significant (*p* < 0.05) diminution of Human Leukocyte Antigen—DR Isotype (HLA-DR) expression in the intermediate and non-classical monocyte populations, but not in classical monocytes (Fig. 2D).

The increase in the percentage of non-classical and intermediate monocytes observed 1–2 days post fistuloplasty could have been due to either a decrease in the number of classical monocytes or an increase in CD16 expressing monocytes. To explore this further, another flow cytometry experiment was carried out using absolute counting beads on whole blood in a separate cohort of patients to enable the exact quantification of the number of cells within each monocyte subpopulation. The previous experiment had also raised the question as to whether this observed shift in monocyte population occurred immediately post fistuloplasty and so the experiment was carried out as a time course of up to three hours post fistuloplasty on the day of their procedure, which is when patients would usually be discharged from the ward.

**Figure 3:**
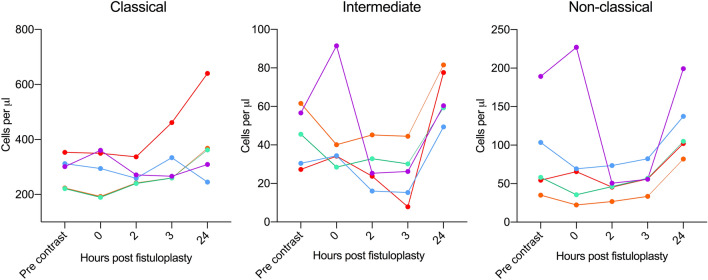
Absolute numbers of monocyte subsets in whole blood from multiple time points before, during, and after a fistuloplasty procedure. Graphs showing the absolute numbers of classical, intermediate and non-classical monocytes prior to the administration of contrast, immediately, 2, 3 and 24 h post fistuloplasty in five donors.

There was no evidence of a reduction in the absolute number of classical monocytes at any time point. The absolute number of non-classical monocytes increased at 24, but not 3 h, post fistuloplasty (Fig. 3). The intermediate monocytes decreased 3 h post fistuloplasty. The number of intermediate monocytes was higher than baseline at day 1. This showed that the increase in the proportion of non-classical and intermediate monocytes seen at day 1–2 (Fig. 2B,C) was due to an increase in the numbers in the CD16 expressing subsets, and not due to a decrease in the number of classical monocytes.

### Associations of monocytes with post intervention primary patency

We explored associations between monocyte subsets and PIPP outcome as this could suggest a functional significance to the changes observed. In our sample, we found evidence of an association between non-classical monocytes and time to loss of PIPP using a univariate cox regression analysis: higher pre-fistuloplasty levels were associated with a slightly greater risk of reaching the endpoint (HR 1.081, 95% CI 1.001–1.167, *p* = 0.046); and the direction of this association was also observed with post-fistuloplasty levels of non-classical monocytes (HR 1.087, 95% CI 0.999–1.183, *p* = 0.053) (Table 2). There was no evidence of an association between classical and intermediate pre and post levels with the outcome in our sample. There was also no association in the change (pre versus post) in monocytes for any subset.

**Table 2:** Univariate cox regression models exploring associations between monocyte subset percentages with time to end of post-intervention primary patency outcome.

Monocyte %	HR	95% CI	*P* value
Pre fistuloplasty classical	0.974	0.934–1.015	0.207
Post fistuloplasty classical	0.986	0.946–1.028	0.501
Pre fistuloplasty intermediate	1.018	0.953–1.087	0.592
Post fistuloplasty intermediate	0.994	0.932–1.059	0.847
Pre fistuloplasty non-classical	1.081	1.001–1.167	0.046
Post fistuloplasty non-classical	1.087	0.999–1.183	0.053
Change in classical monocytes	1.023	0.960–1.090	0.472
Change in intermediate monocytes	0.956	0.871–1.048	0.338
Change in non-classical monocytes	0.979	0.816–1.176	0.979

n = 20 pre fistuloplasty patients, n = 20 post fistuloplasty patients.

### Changes in soluble factors following a fistuloplasty

**Figure 4:**
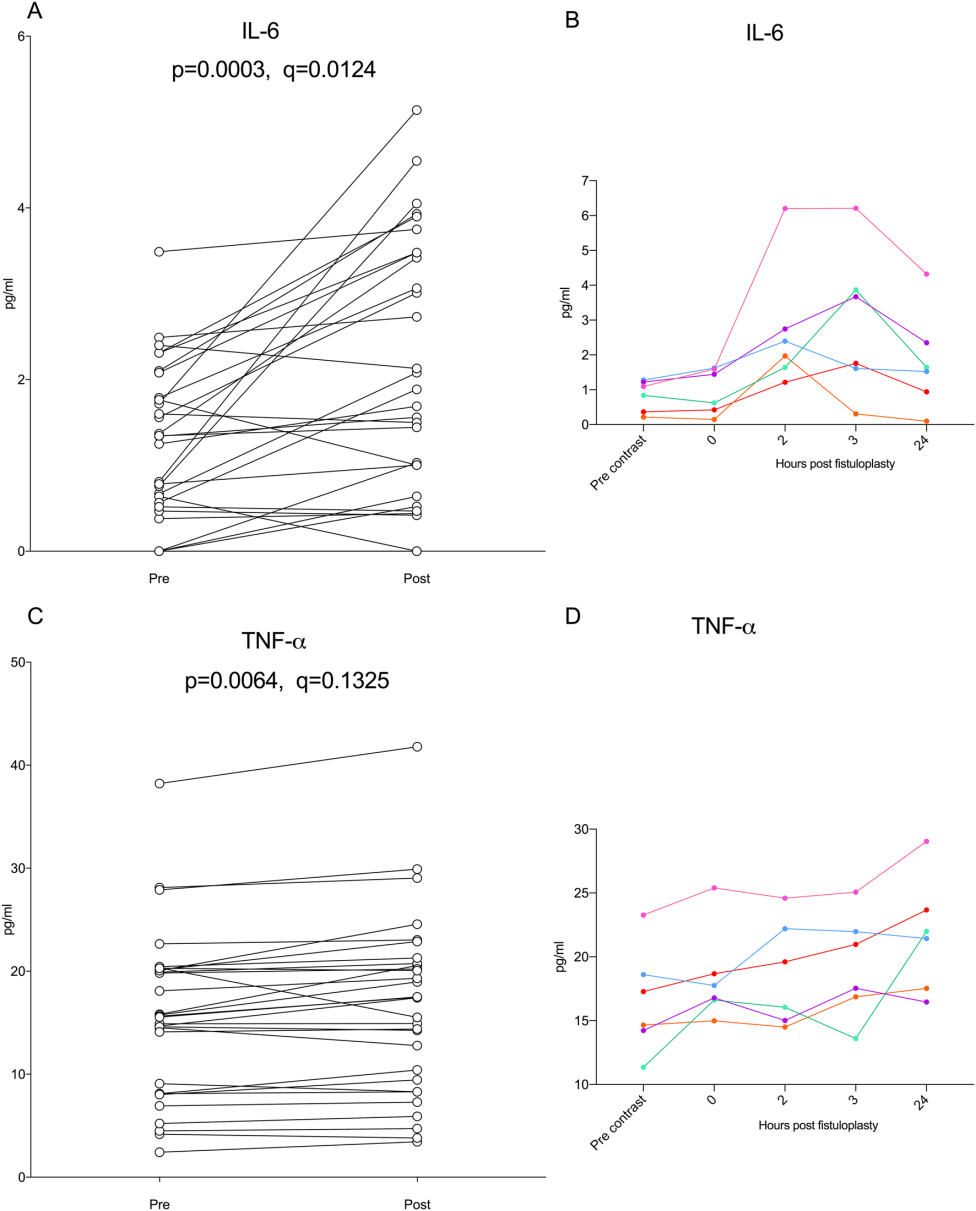
IL-6 and TNF-α following a fistuloplasty procedure. (**A**) IL-6 levels in paired patient plasma samples before and 1–2 days after their fistuloplasty. n = 30 paired samples. Data was analysed using a paired t test. (**B**) Plasma protein levels of IL-6 through the course of the fistuloplasty procedure. n = 6 patients. (**C**) TNF-α levels in paired patient plasma samples before and 1–2 days after their fistuloplasty. n = 30 paired samples. (**D**) Plasma protein levels of TNF-α through the course of the fistuloplasty procedure. n = 6 patients.

We went on to investigate changes in soluble factors after a fistuloplasty procedure. We measured levels of 41 different soluble factors in the plasma of 54 patients who donated pre-procedure samples, and also tested paired post-procedure samples from 30 of these 54 patients. There was no observable pattern for most soluble factors post fistuloplasty procedure and this is shown in the supplementary material (Figure S2). After correcting for false discovery, using the method of Benjamini, Krieger and Yekutieli, only IL-6 (Fig. 4A) showed a statistically significant increase (q = 0.012). The increase in TNF-α (Fig. 4C) was significant when assessed using a paired t test (*p* = 0.006), but the difference was not significant after correction for false discovery (q = 0.1325). We examined changes in both IL-6 and TNF-α in a time-course study with a separate cohort of 6 patients. In all patients, IL-6 peaked between 2–3 h post procedure (Fig. 4B). The plasma levels of TNF-α continued to rise in 4 out of 6 patients up to 24 h post fistuloplasty (Fig. 4D). The observation of an increase in TNF-α in this time course study using a separate patient cohort (Fig. 4C) suggests that the increase at day 1–2 in the larger cohort of experiments (Fig. 4C) was a true result and was not observed by chance.

We investigated for associations between the outcome post fistuloplasty and levels of soluble factors (Table S3). We assessed the levels of soluble factors before (n = 54) and after (n = 30) the procedure, in addition to the change in levels (n = 30). *P* values of < 0.05 were found for pre-procedure M-CSF and MPO. However, q values were not significant after correcting for false discovery (Table S3).

## Discussion

In this report, we have demonstrated that there is a systemic inflammatory response to a localised angioplasty treatment of a specific venous segment. We have shown that there is an increase in circulating proinflammatory cytokines, most notably IL-6 accompanied by an increase in the absolute number of non-classical and intermediate monocytes. Furthermore, within a modest patient cohort we found that higher levels of non-classical monocytes were associated with greater risk of reaching PIPP, suggesting a functional significance to these changes. As frequently observed in clinical studies there was considerable variability within groups. The timing of blood samples (see “Methods” section) minimised any effect of dialysis on the results.

Previous studies have explored circulating monocytes in the context of angioplasty for peripheral arterial disease, but there has been a paucity of data on circulating monocyte phenotype following angioplasty of an arteriovenous fistula. One study in peripheral arterial disease did not demonstrate a change in the relative numbers in monocyte subsets following an angioplasty^16^, however, the paired post angioplasty sample was taken only 15 min after the procedure whilst our study suggests that this shift in monocyte subsets following angioplasty of an arteriovenous fistula occurs after more than three hours’.

The increase in non-classical and intermediate monocytes is likely to be the result of systemic inflammation resulting from vein trauma. Indeed, a number of other studies have shown an increase in circulating CD16+ monocytes in conditions associated with systemic disease and inflammation. In a series of severely injured trauma patients, the majority had an increase in intermediate monocytes^17^. An increase in intermediate monocytes has also been observed in rheumatoid arthritis, after a myocardial infarction and after a stroke^18–20^.

There is evidence of a link between monocyte subsets and PIPP in our sample, but this does not necessarily mean it is causative and we acknowledge the effect was small. In vitro co-culture studies have found that human non‐classical monocytes induce endothelial cell proliferation in a VEGFA‐dependent manner, and that Ly6C^low^ monocytes, the murine equivalent of non-classical monocytes, play a key role in endothelial regeneration after carotid artery injury^21^. It is therefore biologically plausible, that the increase in non-classical monocytes we observed is important in the repair processes that follow the trauma inflicted on the vessel wall during a fistuloplasty. This may, however, have subsequently led to an over active wound healing repair response that could lead to the pathology of restenosis. Indeed, in one report, the proportion of intermediate monocytes associated with restenosis following a femoral-popliteal angioplasty^22^, thus suggesting parallels with the current report. Moreover, the proportions of monocyte subsets have shown correlations with outcome in numerous other contexts including in strokes and cardiovascular events in chronic kidney disease^18,23–26^.

We also observed a decrease in the expression of Human leukocyte antigen-DR (HLA-DR), an MHC class II cell surface molecule on non-classical and intermediate monocytes. Several reports have shown reduced expression of HLA-DR in critically ill patients and that the level of expression associates with immunosuppression, outcome and death^27–30^. Whilst these reports assessed HLA-DR expression on total monocytes, in a study where HLA-DR expression on non-classical and intermediate monocyte subsets was measured, similar changes were found^31^. The functional relevance of the decrease in HLA-DR expression we observed in non-classical and intermediate monocytes post-fistuloplasty is not clear. Nonetheless, it does suggest a deactivation, and switch away from an inflammatory phenotype, which may be further relevant to an over active repair response.

It is possible that IL-6 or other cytokines released at the time of the angioplasty play a role in initiating neointimal hyperplasia. In mice, IL-6 deficiency protected from neointimal formation after femoral artery wire-induced injury^32^. Although these results from the artery of a mouse may not translate to the vein of a human, they make it plausible that IL-6 may have a role in promoting neointimal hyperplasia in arteriovenous fistulas. The lack of association of IL-6 with outcome in our study may have been due to the limited cohort size. Furthermore, a lack of association of changes in circulating IL-6 with outcome would not necessarily exclude a pathogenic role because the magnitude of the rise in an individual patient may not be proportional to the effect of IL-6 in that patient.

The sequential release of classical, intermediate and non-classical human monocyte subsets after an endotoxin challenge has been shown^33^. This establishes a link between a systemic inflammatory insult and changes in monocyte subsets. In the context of our data, we do not suggest that IL-6 is specifically causing the changes in monocyte subsets, but it is a marker of the systemic inflammatory response that has occurred. The specific mediators causing effects on monocytes remain to be defined. We acknowledge that further work will be needed to confirm the changes we have seen in monocyte subsets and IL-6 in another larger patient cohort. We also acknowledge that the changes in IL-6 concentration were fairly modest. Anti-IL-6 therapies are readily available and are an attractive alternative to high dose corticosteroids. They have been shown to be effective for a range of systemic inflammatory states, including severe COVID disease^34^. Data from patients with rheumatoid arthritis shows that tociluzimab is safe and an increased infection risk was only seen in patients also given methotrexate^35^. An additional potential strategy would be to target non-classical and intermediate monocytes. A monoclonal antibody against macrophage colony stimulating factor has been shown to reduce both non-classical and intermediate subsets^36^.

The benefit of currently available interventions to improve outcomes after a fistuloplasty are uncertain. Paclitaxel-coated balloons offered benefit in some studies but not others although these differences may be related to the use of different devices^9–11^. The data presented here suggest that there is a systemic response to a fistuloplasty procedure and this may limit the benefit of local treatments. We should continue to investigate therapies to increase the survival of arteriovenous fistulas and, based on this study, IL-6 and monocyte subsets may be promising future targets.

## Methods

### Sample collection and processing

For the analysis of changes occurring 1–2 days post fistuloplasty, blood was processed from 54 different patients undergoing a fistuloplasty procedure from 3 different trusts across London, (Guy's and St Thomas' NHS Foundation Trust, Royal Free London NHS Foundation Trust and Barts Health NHS Trust). Patients underwent a plain balloon fistuloplasty procedure as specified in the protocol for the PAVE trial^37^. Patients included had consented to the PAVE trial but were not randomised and given a study treatment. In all cases there was a clinical indication for the fistuloplasty. None of the fistulas were thrombosed at the time of intervention. We recorded if patients had a previous radiological intervention. All blood samples were drawn into ethylenediaminetetraacetic acid tubes. Pre-fistuloplasty blood samples were taken with at least 10 h since any previous dialysis session. Post fistuloplasty procedure blood was taken from 30 patients 1–2 days post procedure and was taken at their next dialysis session (obtained pre-dialysis).

Blood was processed into plasma and PBMCs within 8 h of being drawn. For plasma isolation, blood was initially centrifuged at 1000×g for 5 min. Plasma was pipetted off the anti-coagulated blood layer and centrifuged further at 1000×g for 5 min further to isolate plasma. The plasma was stored at − 80 °C. PBMCs of patients were isolated by Ficoll-Paque (GE Healthcare) density gradient centrifugation and were initially stored at − 80 °C before being transferred to liquid nitrogen.

For the time-course study, samples were taken from 6 patients at Guy's and St Thomas' NHS Foundation Trust. The patients all had a clinical indication for a fistuloplasty which was performed as indicated above. Blood was drawn into ethylenediaminetetraacetic acid tubes at the time points indicated and used for plasma isolation (as above) or for whole blood flow cytometry.

### Flow cytometry analysis of antigen expression on peripheral blood cells

A total of 500,000 PBMCs were resuspended in 1 ml of PBS and stained with 1 µl of Fixable Viability Dye eFluor™ 450 (ThermoFisher) for live dead staining. Cells were then washed and then stained for 30 min at room temperature in the dark with 100 µl fluorochrome-conjugated monoclonal antibodies. Samples were analysed using a Becton Dickinson LSRFortessa™ with FACSDiva software (Becton Dickinson). At least 10,000 events were collected per sample and data were analysed using FlowJo software (TreeStar).

For analysis of the monocyte populations, the following fluorochrome-conjugated monoclonal antibodies were used: anti-CD14-APC (clone M5E2, Becton Dickinson), anti-CCR2-FITC (clone K036C, Biolegend), anti-CD3-PerCP-C5.5 (clone OKT3, Biolegend), anti-CD19-PerCP-C5.5 (clone SJ25C1, Biolegend), anti-γδ TCR-PerCP-C5.5 (clone GL3, Biolegend), anti-NKp46-PerCP-C5.5 (clone GL3, Biolegend), anti-CD66b-PerCP-C5.5 (cloneG10F5, Biolegend), anti-CD16-BV510 (clone 3G8, Becton Dickinson), anti-HLA-DR-PE (clone L243, Biolegend), anti-CD11b-Alexa Fluor 700 (clone ICRF44, Biolegend).

Singlets were gated for viability and then gated as potential monocytes by drawing a generous gate based on forward and side scatter properties. CD3+, CD19+, CD3+, γδ+CD66b+ and NKp46+ cells were dumped and the remaining cells were then gated based on monocyte’s characteristic morphology when gated for CD14 and CD16. Contaminating cells were further removed by gating HLA-DR with CD16. Monocyte subsets were then gated based on CD14 and CD16 expression. Monocyte subsets were further confirmed to be classic, intermediate and non-classical monocytes based on their levels of CCR2, HLA-DR and CD11b expression. Pre and post samples from a given patient were always analysed together and the experiment was run in two batches of ten paired samples.

### Flow cytometry analysis of antigen expression in whole blood

For the analysis of monocyte populations in whole blood, 100 μl of EDTA blood was pipetted into Absolute Counting Tubes (Becton Dickinson) and incubated at room temperature for 20 min protected from the light with the following fluorochrome-conjugated monoclonal antibodies: anti-CD14-Alexa Fluor 647 (clone M5E2, Biolegend), anti-CD16-BV510 (clone 3G8, Biolegend), anti-CD66b-Alexa Fluor 700 (clone G10F5, Biolegend), anti-NKp46-FITC (clone 9E2, Biolegend), anti-CD11b-FITC (clone ICRF44, Biolegend), anti-CD3-FITC (clone UCHT1, Biolegend), anti-CD19-FITC (clone H1B19, Becton Dickinson), anti-HLA-DR-BUV395 (clone G46-6, Becton Dickinson), anti-CCR2-BV421 (clone 48,607, Becton Dickinson) and anti-CD45-BV786 (clone HI30, Becton Dickinson). After staining, the blood was incubated for 15 min at room temperature in the dark with 1-step fix/lyse solution (ThermoFisher). Stained fixed and lysed blood was stored at 4 °C in the dark before being run on an LSRFortessa™ with FACsDIVA software (Becton Dickinson) the next day. At least 5000 bead events were collected from each fully stained tube at the low acquiring speed and data was analysed using FlowJo software (TreeStar).

### Luminex assays and ELISAs

The following plasma proteins were measured with MagPlex Luminex assays (Bio-techne) using a Luminex™ FLEXMAP 3D™ (Luminex): Angiogenin, Angiopoietin-1, Angiopoietin-2, BDNF, CD30, CD40 Ligand, CTACK, Flt3 Ligand, Eotaxin, HGF, IL-1ra, IL2Rα, IL-6, IL-16, IL-17A, IL-18, MCP-1, MIP-3α, MIF, MIG, MIP-3β, MMP-1, MMP-8, MMP-12, MMP-13, PDGF-AA, PF4, RAGE, RANTES, SCGF, S100B, S100A9, TNF-α. Adiponectin, BDNF, calprotectin, CRP, M-CSF, MPO, PDGF-BB, TfR, VEGF were measured with Duoset ELISAs (Bio-techne) according to the manufacturer’s instructions in 96 well flat bottom microplates (Nunc). Plasma samples were run in duplicates and in all instances, paired pre and post samples were assayed on the same plate, with 20 paired samples run on the first batch and 10 paired samples run on the second batch.

### Ethics approval and consent to participate

Fistuloplasty time course blood samples were collected under an ethical approval from South Central Berkshire B Research Ethics Committee (REC reference: 15/SC/0764). All other blood samples were collected using the ethics for the PAVE clinical trial, approved by London-Chelsea Research Ethics Committee (REC reference: 15/LO/0638). Informed consent was obtained from all participants and all research was performed in accordance with relevant guidelines and regulations and in accordance with the Declaration of Helsinki.

### Statistical analyses

Univariate cox regression analysis was carried out in IBM SPSS statistics (version 26) to explore associations between monocyte subsets or soluble factors and PIPP. PIPP was defined as ending when there was a radiological intervention, a surgical intervention or a thrombosis anywhere in the access circuit. PIPP also ended when the fistula was abandoned. Participants were censored if they had a kidney transplant, switched to peritoneal dialysis, died or had not lost patency at the end of follow up. All other statistics were carried out using GraphPad Prism (GraphPad software version 8.1) using a paired t-test.

### Supplementary Information

Supplementary Information 1.

## Data Availability

Raw data will be made available to any qualified researcher with a reasonable request submitted to the corresponding author Michael Robson (Michael.robson@kcl.ac.uk).
