# Author Correction: Circulating Interleukin‑6 and CD16 positive monocytes increase following angioplasty of an arteriovenous fistula

**DOI:** 10.1038/s41598-022-22213-0

**Published:** 2022-10-31

**Authors:** Seran Hakki, Emily J. Robinson, Michael G. Robson

**Affiliations:** 1 School of Immunology and Microbial Sciences, Faculty of Life Sciences and Medicine, King’s College London, Guy’s Hospital, London, SE1 9RT UK; 2 School of Population Health and Environmental Sciences, Faculty of Life Sciences and Medicine, King’s College London, London, UK

Correction to: *Scientific Reports* https://doi.org/10.1038/s41598-022-05062-9, published online 26 January 2022

The Supplementary Information file published with this Article contained an error in the legend of Table S3.

“Table S3: Univariate cox regression analysis showing the association soluble factors with post intervention primary patency. Table showing the p values of the associations between the levels of each pre fistuloplasty protein, post fistuloplasty protein and change in protein level (calculated by subtracting post fistuloplasty with the pre fistuloplasty plasma protein levels) with either post-intervention primary patency or post-intervention cumulative patency outcome. (n = 54 patients for pre fistuloplasty plasma proteins, n = 30 post fistuloplasty and change in plasma proteins patient samples). Where p values were < 0.05, false discoveries were calculated using the method of Benjamini, Krieger and Yekutieli to give q values.”

now reads:

“Table S3: Univariate cox regression analysis showing the association soluble factors with post intervention primary patency. Table showing the p values of the associations between the levels of each pre fistuloplasty protein, post fistuloplasty protein and change in protein level (calculated by subtracting post fistuloplasty with the pre fistuloplasty plasma protein levels) with post-intervention primary patency patency. Sample numbers were n = 54 for pre-fistuloplasty and n = 30 for post-fistuloplasty and change (post minus pre). Where p values were < 0.05, false discoveries were calculated using the method of Benjamini, Krieger and Yekutieli to give q values which are shown in brackets.”

This error has now been corrected in the Supplementary Information file that accompanies the original Article.

## Supplementary Information

Supplementary Information.

