# Author Correction: Circulating Interleukin-6 and CD16 positive monocytes increase following angioplasty of an arteriovenous fistula

**DOI:** 10.1038/s41598-023-44596-4

**Published:** 2023-10-26

**Authors:** Seran Hakki, Emily J. Robinson, Michael G. Robson

**Affiliations:** 1 School of Immunology and Microbial Sciences, Faculty of Life Sciences and Medicine, King’s College London, Guy’s Hospital, London, SE1 9RT UK; 2 School of Population Health and Environmental Sciences, Faculty of Life Sciences and Medicine, King’s College London, London, UK

Correction to: *Scientific Reports* 10.1038/s41598-022-05062-9, published online 26 January 2022

The original version of this Article contained errors.

Firstly in the Results section under the sub-heading “Changes in soluble factors following a fistuloplasty,” the sentence:

“We measured levels of 41 different soluble factors in the plasma of 54 patients who donated pre-procedure samples, of which 30 gave paired post-procedure samples.”

now reads:

“We measured levels of 41 different soluble factors in the plasma of 54 patients who donated pre-procedure samples, and also tested paired post-procedure samples from 30 of these 54 patients.”

Secondly in the Methods section under the subheading “Sample collection and processing,” a sentence was omitted in the paragraph:

“For the analysis of changes occurring 1–2 days post fistuloplasty, blood was processed from 54 different patients undergoing a fistuloplasty procedure from 3 different trusts across London, (Guy's and St Thomas' NHS Foundation Trust, Royal Free London NHS Foundation Trust and Barts Health NHS Trust). Patients underwent a plain balloon fistuloplasty procedure as specified in the protocol for the PAVE trial^37^. In all cases there was a clinical indication for the fistuloplasty. None of the fistulas were thrombosed at the time of intervention. We recorded if patients had a previous radiological intervention. All blood samples were drawn into ethylenediaminetetraacetic acid tubes. Pre-fistuloplasty blood samples were taken with at least 10 h since any previous dialysis session. Post fistuloplasty procedure blood was taken from 30 patients 1–2 days post procedure and was taken at their next dialysis session (obtained pre-dialysis).”

It now reads:

For the analysis of changes occurring 1–2 days post fistuloplasty, blood was processed from 54 different patients undergoing a fistuloplasty procedure from 3 different trusts across London, (Guy's and St Thomas' NHS Foundation Trust, Royal Free London NHS Foundation Trust and Barts Health NHS Trust). Patients underwent a plain balloon fistuloplasty procedure as specified in the protocol for the PAVE trial^37^. Patients included had consented to the PAVE trial but were not randomised and given a study treatment. In all cases there was a clinical indication for the fistuloplasty. None of the fistulas were thrombosed at the time of intervention. We recorded if patients had a previous radiological intervention. All blood samples were drawn into ethylenediaminetetraacetic acid tubes. Pre-fistuloplasty blood samples were taken with at least 10 h since any previous dialysis session. Post fistuloplasty procedure blood was taken from 30 patients 1–2 days post procedure and was taken at their next dialysis session (obtained pre-dialysis).

Thirdly in the same section there was a paragraph that was missing. It now reads:

“For the time-course study, samples were taken from 6 patients at Guy's and St Thomas' NHS Foundation Trust. The patients all had a clinical indication for a fistuloplasty which was performed as indicated above. Blood was drawn into ethylenediaminetetraacetic acid tubes at the time points indicated and used for plasma isolation (as above) or for whole blood flow cytometry.”

Fourthly, in the Methods section, under the subheadings ‘Statistical analyses’,

Univariate cox regression analysis was carried out in IBM SPSS statistics (version 26) to explore associations between monocyte subsets measured pre and post intervention and PIPP, defined here as the time from when patency is restored following a successful intervention to the access circuit to when the access circuit requires a re-intervention. All other statistics was carried out using GraphPad Prism (GraphPad software version 8.1) using a paired t-test.

now reads:

Univariate cox regression analysis was carried out in IBM SPSS statistics (version 26) to explore associations between monocyte subsets or soluble factors and PIPP. PIPP was defined as ending when there was a radiological intervention, a surgical intervention or a thrombosis anywhere in the access circuit. PIPP also ended when the fistula was abandoned. Participants were censored if they had a kidney transplant, switched to peritoneal dialysis, died or had not lost patency at the end of follow up. All other statistics were carried out using GraphPad Prism (GraphPad software version 8.1) using a paired t-test.

And Finally,

The Supplementary Information 1 file published with this Article contained an error where in the far-right column of Table S1, some of the reasons for censoring were not shaded grey. The original Supplementary Information 1 file is provided below.

This error has now been corrected in the Supplementary Information 1 file that accompanies the original Article.

The original Article has been corrected.

### Supplementary Information

Supplementary Information 1.

