# Supplementary material for: Circulating Interleukin-6 and CD16 positive monocytes increase following angioplasty of an arteriovenous fistula
Source: Sci Rep. 2022 Jan 26;12:1427. doi: 10.1038/s41598-022-05062-9 (PMC8792046; doi:10.1038/s41598-022-05062-9)
Supplement: Supplementary file 1 — Supplementary Information 1. [file 41598_2022_5062_MOESM1_ESM.pdf]

**Supplementary Material**

**Table S1. Demographic features and post intervention primary patency data**

**Table S2. Soluble factors measured following a fistuloplasty (p and q values)**

**Figure S1. Soluble factors measured following a fistuloplasty (concentrations)**

**Table S3: Univariate cox regression analysis showing the association soluble factors with post intervention primary patency**

**Table S1.** Table showing the demographic features and post intervention primary patency information of the patients within this study. “Pre & Post Monocytes” denotes patients from which PBMC samples were used for investigating changes in monocyte phenotype post fistuloplasty. “Pre & Post Soluble Factors” denotes patients from which plasma samples were used for investigating changes in soluble factors post fistuloplasty. “RC” denotes radio-cephalic, “BC” denotes brachiocephalic and “BVT” denotes basilic vein transposition fistula. “PIPP” denotes post intervention primary patency. Patients were followed up for a minimum of one year or until the end of PIPP or censoring.

| Pre & Post Monocytes | Pre & Post Soluble Factors | Gender | Age (years) | Ethnicity | Type of Native Fistula | Fistula Age (years) | Previous Thrombosis | Previous Fistuloplasty | Reason for intervention | Fistula in use? | Medication | N. Days of follow up | Reason for the end of post intervention primary patency or for censoring (shaded grey) |
|---|---|---|---|---|---|---|---|---|---|---|---|---|---|
| ✓ | ✓ | F | 56 | Asian | BVT | 4.80 | N | Y | 7 | Y | N | 1177 | Kidney transplant |
| ✓ | ✓ | F | 67 | White | BC | 4.51 | N | Y | 6 | Y | A, S | 84 | Decision to Abandon |
| ✓ | ✓ | M | 83 | Black | BVT | 0.21 | N | N | 2 | N | A, S | 114 | Thrombosed |
| ✓ | ✓ | F | 76 | White | BC | 5.86 | N | Y | 7 | Y | W,S | 624 | Decision to Abandon |
| ✓ | ✓ | M | 20 | Asian | RC | 1.76 | N | N | 2 | Y | N | 98 | Thrombosed |
| ✓ | ✓ | M | 60 | Black | RC | 0.08 | N | N | 2 | N | P,T,M | 280 | Radiological Procedure |
| ✓ | ✓ | M | 56 | Black | BC | 0.36 | N | N | 1 | Y | N | 109 | Thrombosed |
| ✓ | ✓ | M | 82 | Black | BVT | 4.82 | N | Y | 4 | Y | P | 562 | Radiological Procedure |
| ✓ | ✓ | F | 70 | White | BC | 1.19 | N | N | 2 | Y | S | 489 | Thrombosed |
| ✓ | ✓ | M | 68 | Black | BC | 0.54 | N | N | 2 | N | A, S | 921 | Radiological Procedure |
| ✓ | ✓ | M | 73 | Black | BC | 5.83 | N | N | 4 | Y | A,W, S | 158 | Thrombosed |
| ✓ | ✓ | F | 49 | Black | BVT | 1.04 | N | Y | 2 | Y | N | 501 | Radiological Procedure |
| ✓ | ✓ | F | 55 | Black | BC | 2.32 | N | N | 1 | Y | A, | 100 | Radiological Procedure |
| ✓ | ✓ | F | 37 | White | RC | 0.41 | N | N | 6 | Y | N | 140 | Radiological Procedure |
| ✓ | ✓ | M | 52 | Black | RC | 4.47 | N | N | 6 | Y | A | 823 | End of Follow Up |
| ✓ | ✓ | F | 33 | White | BC | 2.84 | N | Y | 4 | Y | N | 781 | Radiological Procedure |
| ✓ | ✓ | M | 53 | White | BVT | 0.59 | N | N | 2 | Y | N | 739 | Death |
| ✓ | ✓ | F | 38 | Asian | BVT | 1.10 | N | N | 3 | Y | N | 204 | Radiological Procedure |
| ✓ | ✓ | M | 88 | White | BC | 1.20 | N | N | 3 | Y | S, P | 291 | Thrombosed |
| ✓ | ✕ | F | 63 | White | BC | 0.64 | N | N | 6 | Y | A. S | 294 | Radiological Procedure |
| ✕ | ✓ | M | 62 | White | RC | 12.75 | N | N | 5 | Y | S | 230 | Death |
| ✕ | ✓ | F | 59 | Black | BC | 0.24 | N | N | 6 | Y | A | 109 | Thrombosed |
| ✕ | ✓ | M | 68 | Black | RC | 3.52 | N | N | 1 | Y | N | 1353 | End of Follow Up |
| ✕ | ✓ | M | 70 | White | BC | 4.02 | N | N | 6 | Y | N | 52 | Radiological Procedure |
| ✕ | ✓ | M | 60 | Asian | BVT | 0.31 | N | N | 2 | Y | A | 123 | Surgical Intervention |
| ✕ | ✓ | M | 79 | White | BC | 1.85 | Y | Y | 2 | Y | A, S | 253 | Decision to Abandon |
| ✕ | ✓ | M | 67 | White | BC | 1.40 | N | N | 3 | Y | A, S | 284 | Surgical Intervention |
| ✕ | ✓ | F | 41 | Black | BC | 4.51 | N | Y | 3 | Y | N | 514 | Kidney transplant |
| ✕ | ✓ | F | 62 | Black | BC | 2.31 | N | N | 7 | N | N | 371 | Radiological Procedure |
| ✕ | ✓ | M | 62 | Black | RC | 0.627 | N | N | 2 | Y | N | 751 | End of Follow Up |

| | | | | | | | | | | | | | |
|---|---|---|---|---|---|---|---|---|---|---|---|---|---|
| ✕ | ✓ | F | 71 | Black | BC | 1.22 | N | N | 2 | Y | S | 71 | Thrombosed |
| ✕ | ✕ | F | 48 | Black | BVT | 2.72 | N | N | 4 | Y | N | 511 | Radiological Procedure |
| ✕ | ✕ | M | 48 | Black | BVT | 0.25 | N | N | 2 | N | S | 316 | Radiological Procedure |
| ✕ | ✕ | F | 70 | Asian | BC | 4.40 | N | Y | 7 | Y | A, S | 82 | Decision to Abandon |
| ✕ | ✕ | F | 45 | Black | RC | 1.81 | N | Y | 6 | Y | A | 252 | Radiological Procedure |
| ✕ | ✕ | M | 75 | White | BC | 1.44 | N | N | 7 | N | N | 84 | Decision to Abandon |
| ✕ | ✕ | M | 61 | Black | BVT | 0.98 | N | N | 2 | Y | N | 124 | Thrombosed |
| ✕ | ✕ | F | 49 | Black | BVT | 4.12 | N | Y | 6 | Y | A | 48 | Kidney transplant |
| ✕ | ✕ | M | 57 | Black | RC | 1.68 | N | N | 6 | Y | N | 180 | Surgical Intervention |
| ✕ | ✕ | M | 52 | Black | RC | 0.58 | N | N | 6 | N | A,S | 339 | Thrombosed |
| ✕ | ✕ | F | 71 | Asian | BVT | 0.53 | N | N | 6 | Y | A | 50 | Radiological Procedure |
| ✕ | ✕ | M | 31 | White | BC | 1.49 | N | N | 2 | N | N | 70 | Kidney transplant |
| ✕ | ✕ | M | 67 | White | RC | 3.41 | N | N | 6 | Y | A, S | 623 | End of Follow Up |
| ✕ | ✕ | M | 76 | Asian | BVT | 0.91 | N | N | 6 | Y | A, S | 215 | Decision to Abandon |
| ✕ | ✕ | F | 74 | Black | BC | 3.85 | N | Y | 6 | Y | A, S | 187 | Decision to Abandon |
| ✕ | ✕ | M | 61 | Black | BC | 1.48 | N | N | 3 | Y | C | 294 | Radiological Procedure |
| ✕ | ✕ | F | 49 | White | BC | 0.35 | N | N | 4 | Y | N | 366 | Transplant |
| ✕ | ✕ | M | 55 | Black | BC | 7.07 | Y | Y | 6 | Y | N | 138 | Transplant |
| ✕ | ✕ | M | 71 | Black | BVT | 2.54 | N | N | 2 | Y | S | 371 | End of Follow Up |
| ✕ | ✕ | F | 71 | Asian | BVT | 1.11 | N | N | 3 | Y | NA | 96 | Decision to Abandon |
| ✕ | ✕ | M | 71 | Black | RC | 0.98 | N | N | 7 | Y | NA | 460 | Radiological Procedure |
| ✕ | ✕ | M | 59 | White | RC | 0.95 | N | N | 6 | Y | NA | 199 | Death |
| ✕ | ✕ | M | 59 | White | BC | 0.60 | N | N | 2 | Y | NA | 389 | End of Follow Up |
| ✕ | ✕ | F | 48 | White | RC | 5.56 | N | N | 1 | Y | NA | 149 | Surgical Intervention |

Y= Yes, N=No. **Reason for intervention**; 1=inadequate dialysis, 2=decreased flow, 3=prolonged bleeding, 4=raised venous pressure, 5=decreased arterial pressure, 6=needling difficulties, 7=other. **Medication**; N=not on antiplatelets, statins, warfarin, prednisolone, or immunosuppressants. A=antiplatelet, S=statin, W=warfarin, P=prednisolone, T=tacrolimus, M=mycophenolate, NA=data not available.

**Table S2. Soluble factors measured following a fistuloplasty (p and q values).** Levels of 41 soluble mediators in paired patient plasma samples before and 1-2 days after their fistuloplasty. n = 30 paired samples. p values were calculated using a paired t test. False discoveries were account for using the method of Benjamini, Krieger and Yekutieli to give q values and are given in the table below. Data are shown in figure S1 below.

| Factor | Mean Pre (pg/ml) | Mean Post (pg/ml) | Mean Change (Post - Pre) | p value | q value |
|---|---|---|---|---|---|
| Adiponectin | 11537971.8 | 11021840.8 | -516131.0 | 0.388 | 0.688 |
| Angiogenin | 969494.4 | 922324.2 | -47170.2 | 0.399 | 0.688 |
| Angiopoietin-1 | 11715.8 | 11334.0 | -381.9 | 0.708 | 0.941 |
| Angiopoietin-2 | 6782.5 | 6504.0 | -278.6 | 0.282 | 0.688 |
| BDNF | 8229.6 | 9139.1 | 909.5 | 0.593 | 0.847 |
| Calprotectin | 1480776.1 | 1557052.3 | 76276.1 | 0.670 | 0.925 |
| CD30 | 143.2 | 129.1 | -14.1 | 0.076 | 0.670 |
| CD40 Ligand | 2685.2 | 2370.4 | -314.8 | 0.173 | 0.688 |
| CRP | 4740132.8 | 4722230.6 | -17902.2 | 0.847 | 0.941 |
| CTACK | 811.7 | 817.5 | 5.8 | 0.851 | 0.941 |
| Eotaxin | 697.1 | 707.3 | 10.2 | 0.338 | 0.688 |
| Flt3 Ligand | 90.9 | 96.2 | 5.3 | 0.278 | 0.688 |
| HGF | 516.3 | 514.0 | -2.3 | 0.846 | 0.941 |
| IL-16 | 706.5 | 703.1 | -3.5 | 0.861 | 0.941 |
| IL-17A | 65.6 | 70.1 | 4.4 | 0.496 | 0.761 |
| IL-18 | 408.9 | 397.6 | -11.3 | 0.340 | 0.688 |
| IL-1ra | 1949.4 | 1794.5 | -155.0 | 0.373 | 0.688 |
| IL-2R α | 1858.3 | 1855.3 | -3.1 | 0.956 | 0.966 |
| IL-6 | 1.2 | 2.1 | 0.9 | **0.0003** | **0.012** |
| MCP-1 | 155.0 | 156.2 | 1.2 | 0.919 | 0.951 |
| M-CSF | 139.2 | 150.5 | 11.4 | 0.438 | 0.726 |
| MIF | 178945.3 | 152609.8 | -26335.4 | 0.295 | 0.688 |
| MIG | 1813.8 | 1773.1 | -40.8 | 0.212 | 0.688 |
| MIP-3α | 129.5 | 84.3 | -45.3 | 0.463 | 0.737 |
| MIP-3β | 176.6 | 188.8 | 12.3 | 0.338 | 0.688 |
| MMP1 | 2670.1 | 2542.3 | -127.8 | 0.152 | 0.688 |
| MMP8 | 5094.5 | 5663.3 | 568.8 | 0.312 | 0.688 |
| MMP12 | 50.2 | 50.5 | 0.2 | 0.903 | 0.951 |
| MMP13 | 1359.8 | 1380.8 | 21.0 | 0.291 | 0.688 |
| MPO | 110876.3 | 94430.5 | -16445.8 | 0.040 | 0.548 |
| PDGF-AA | 909.5 | 821.0 | -88.5 | 0.148 | 0.688 |
| PDGF-BB | 4331.1 | 3311.1 | -1020.0 | 0.293 | 0.688 |
| PF4 | 6316647.4 | 4150465.6 | -2166181.9 | 0.194 | 0.688 |
| RAGE | 10245.1 | 10123.0 | -122.1 | 0.783 | 0.941 |
| RANTES | 49453.8 | 62160.7 | 12706.9 | 0.350 | 0.688 |
| S100A9 | 1946.8 | 1454.6 | -492.3 | 0.758 | 0.941 |
| S100B | 881.3 | 862.6 | -18.7 | 0.254 | 0.688 |
| SCGF | 10217.2 | 11275.2 | 1058.0 | 0.081 | 0.670 |
| TfR | 1476515.4 | 1646782.2 | 170266.8 | 0.555 | 0.820 |
| TNF-α | 15.6 | 16.6 | 1.0 | **0.006** | **0.133** |
| VEGF | 208.3 | 210.7 | 2.4 | 0.864 | 0.941 |

**Figure S1. Soluble factors measured following a fistuloplasty (concentrations).** Levels of soluble mediators in paired patient plasma samples before and 1-2 days after their fistuloplasty. n = 30 paired samples. Each symbol pair represents a different patient. The p and q values for each factor are shown above in table S1. Data for IL-6 and TNF$\alpha$ are shown in the main paper.

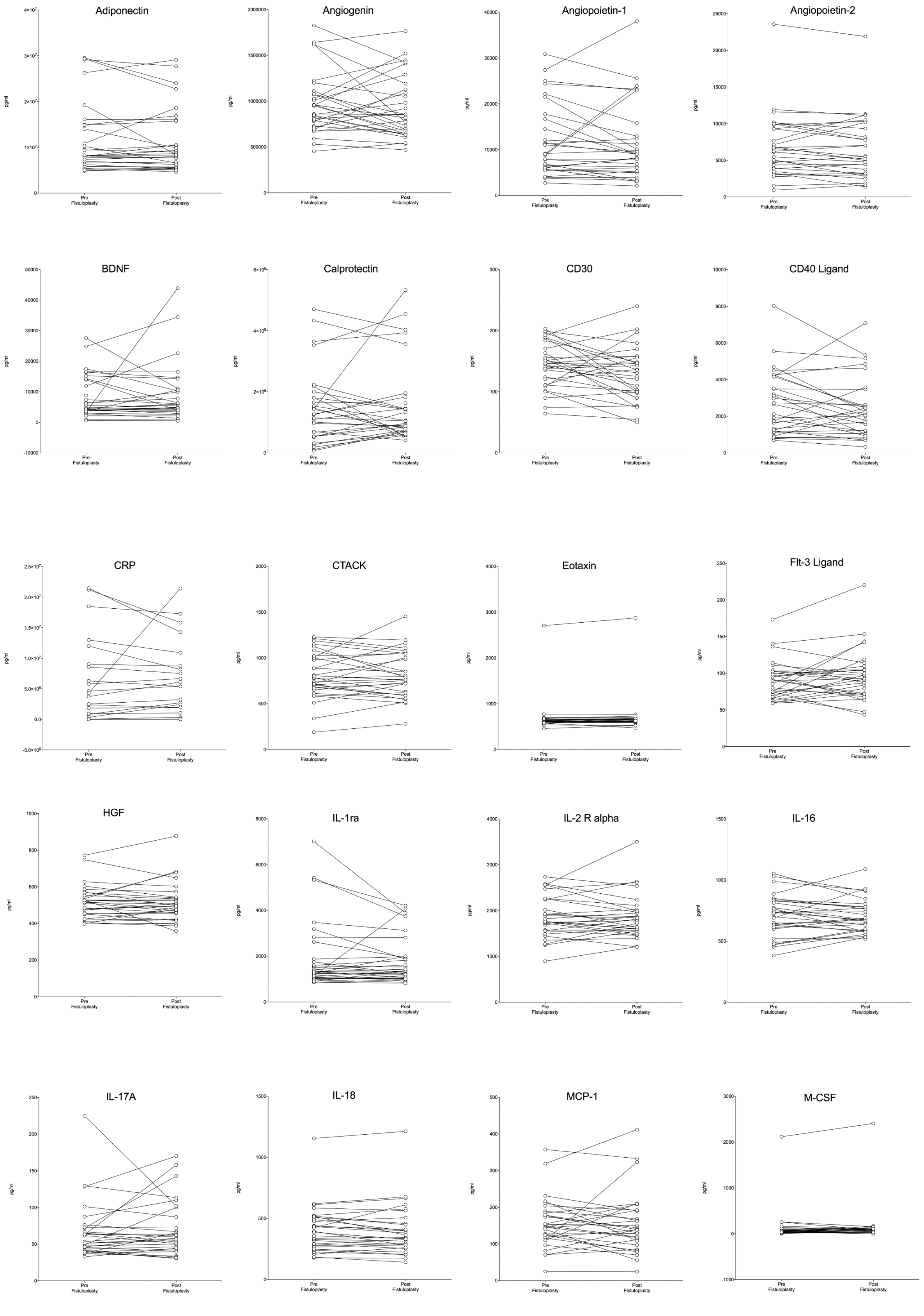

# Figure S1. (continued)

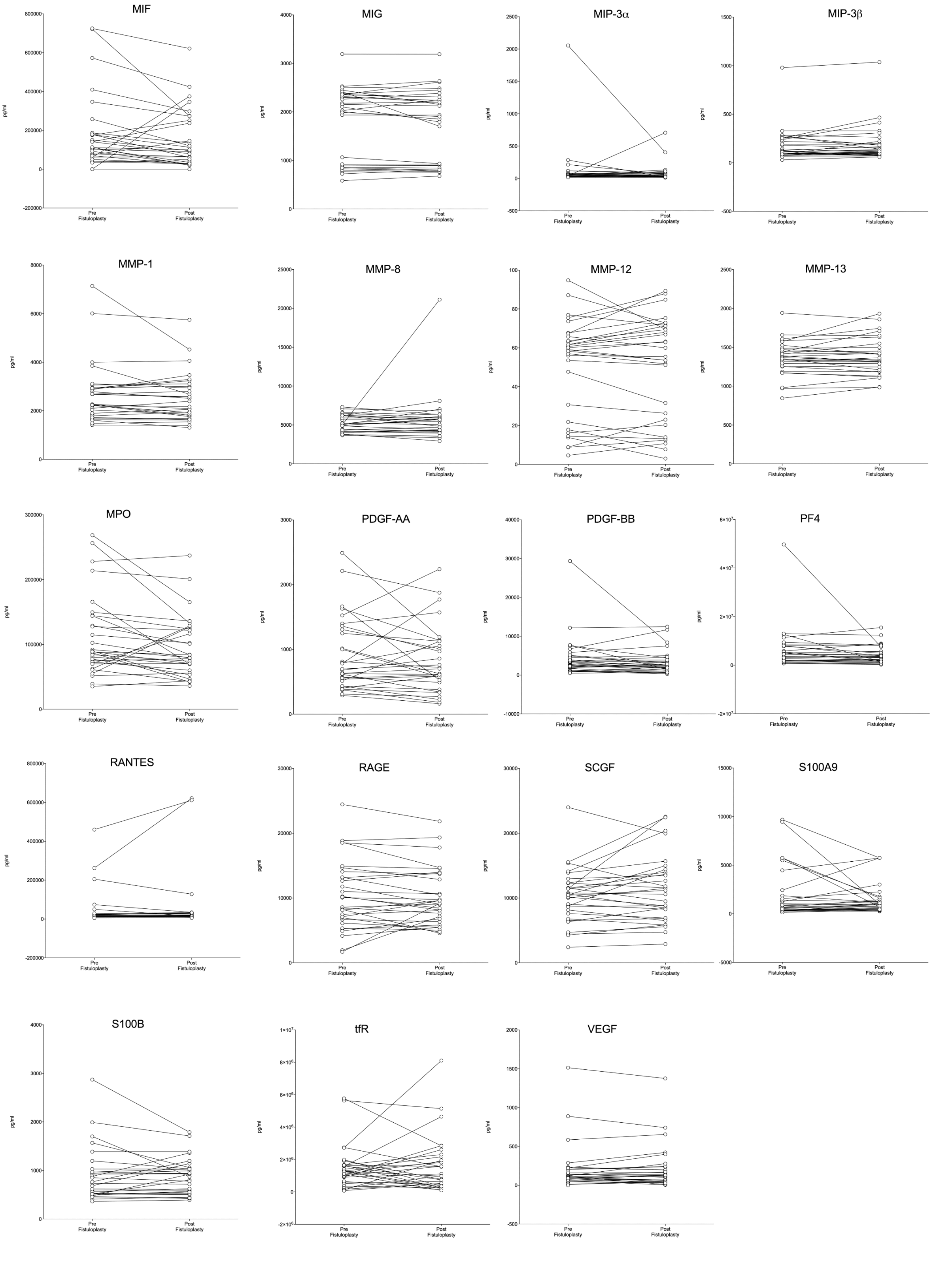

**Table S3: Univariate cox regression analysis showing the association soluble factors with post intervention primary patency**. Table showing the p values of the associations between the levels of each pre fistuloplasty protein, post fistuloplasty protein and change in protein level (calculated by subtracting post fistuloplasty with the pre fistuloplasty plasma protein levels) with post-intervention primary patency patency. Sample numbers were n = 54 for pre-fistuloplasty and n = 30 for post-fistuloplasty and change (post minus pre). Where p values were <0.05, false discoveries were calculated using the method of Benjamini, Krieger and Yekutieli to give q values which are shown in brackets.

| Plasma Protein Time point | Pre Fistuloplasty | | Post Fistuloplasty | | Change (Post minus Pre) | |
|---|---|---|---|---|---|---|
| Protein | Significance | Hazard Ratio | Significance | Hazard Ratio | Significance | Hazard Ratio |
| Adiponectin | 0.377 | 1.000 | 0.223 | 1.000 | 0.833 | 1.000 |
| Angiogenin | 0.124 | 1.000 | 0.671 | 1.000 | 0.852 | 1.000 |
| Angiopoietin-1 | 0.944 | 1.000 | 0.451 | 1.000 | 0.632 | 1.000 |
| Angiopoietin-2 | 0.744 | 1.000 | 0.917 | 1.000 | 0.934 | 1.000 |
| BDNF | 0.982 | 1.000 | 0.642 | 1.000 | 0.691 | 1.000 |
| Calprotectin | 0.257 | 1.000 | 0.496 | 1.000 | 0.625 | 1.000 |
| CD30 | 0.858 | 1.000 | 0.430 | 1.004 | 0.464 | 1.003 |
| CD40 Ligand | 0.806 | 1.000 | 0.370 | 1.000 | 0.932 | 1.000 |
| CRP | 0.644 | 1.000 | 0.461 | 1.000 | 0.931 | 1.000 |
| CTACK | 0.442 | 1.000 | 0.983 | 1.000 | 0.294 | 1.001 |
| Eotaxin | 0.720 | 1.000 | 0.310 | 1.001 | 0.728 | 1.002 |
| Flt3 Ligand | 0.303 | 1.002 | 0.719 | 1.002 | 0.637 | 0.999 |
| HGF | 0.500 | 0.999 | 0.548 | 0.998 | 0.937 | 1.000 |
| IL-16 | 0.570 | 1.000 | 0.654 | 0.999 | 0.451 | 1.002 |
| IL-17A | 0.898 | 1.000 | 0.715 | 0.998 | 0.885 | 0.999 |
| IL-18 | 0.945 | 1.000 | 0.434 | 0.999 | 0.962 | 1.000 |
| IL-1ra | 0.462 | 1.000 | 0.439 | 1.000 | 0.758 | 1.000 |
| IL-2R α | 0.587 | 1.000 | 0.452 | 1.000 | 0.435 | 1.000 |
| IL-6 | 0.431 | 0.871 | 0.216 | 0.838 | 0.160 | 0.769 |
| MCP-1 | 0.330 | 1.002 | 0.567 | 1.001 | 0.940 | 1.000 |
| M-CSF | **0.040** (0.828) | 1.001 | 0.715 | 1.000 | 0.289 | 0.996 |
| MIF | 0.797 | 1.000 | 0.763 | 1.000 | 0.558 | 1.000 |
| MIG | 0.927 | 1.000 | 0.781 | 1.000 | 0.258 | 0.999 |
| MIP-3α | 0.531 | 1.000 | 0.239 | 0.997 | 0.162 | 0.999 |
| MIP-3β | 0.596 | 0.999 | 0.956 | 1.000 | 0.216 | 1.004 |
| MMP1 | 0.899 | 1.000 | 0.962 | 1.000 | 0.637 | 1.000 |
| MMP8 | 0.433 | 1.000 | 0.704 | 1.000 | 0.754 | 1.000 |
| MMP12 | 0.775 | 1.002 | 0.650 | 1.004 | 0.649 | 1.010 |
| MMP13 | 0.870 | 1.000 | 0.657 | 1.000 | 0.153 | 1.003 |
| MPO | **0.003** (0.124) | 1.000 | 0.325 | 1.000 | 0.111 | 1.000 |
| PDGF-AA | 0.976 | 1.000 | 0.407 | 1.000 | 0.894 | 1.000 |
| PDGF-BB | 0.909 | 1.000 | 0.237 | 1.000 | 0.195 | 1.000 |
| PF4 | 0.164 | 1.000 | 0.657 | 1.000 | 0.134 | 1.000 |
| RAGE | 0.518 | 1.000 | 0.112 | 1.000 | 0.949 | 1.000 |
| RANTES | 0.224 | 1.000 | 0.319 | 1.000 | 0.410 | 1.000 |
| S100A9 | 0.273 | 1.000 | 0.159 | 1.000 | 0.445 | 1.000 |
| S100B | 0.277 | 1.000 | 0.754 | 1.000 | 0.505 | 1.001 |
| SCGF | 0.502 | 1.000 | 0.761 | 1.000 | 0.988 | 1.000 |
| TfR | 0.804 | 1.000 | 0.351 | 1.000 | 0.334 | 1.000 |
| TNF-α | 0.198 | 1.025 | 0.338 | 1.022 | 0.894 | 0.981 |
| VEGF | 0.415 | 1.000 | 0.403 | 1.001 | 0.822 | 0.999 |
