# Supplementary material for: Author Correction: Circulating Interleukin‑6 and CD16 positive monocytes increase following angioplasty of an arteriovenous fistula
Source: Sci Rep. 2022 Oct 31;12:18320. doi: 10.1038/s41598-022-22213-0 (PMC9622700; doi:10.1038/s41598-022-22213-0)
Supplement: Supplementary file 1 — Supplementary Information. [file 41598_2022_22213_MOESM1_ESM.pdf]

Supplementary Material

Table S1. Demographic features and post intervention primary patency data

Table S2. Soluble factors measured following a fistuloplasty (p values)

Figure S1. Soluble factors measured following a fistuloplasty (concentrations)

Table S3: Univariate cox regression analysis showing the association soluble factors with post intervention primary patency

Table S1. Table showing the demographic features and post intervention primary patency information of the patients within this study. “Pre & Post Monocytes” denotes patients from which PBMC samples were used for investigating changes in monocyte phenotype post fistuloplasty. “Pre & Post Soluble Factors” denotes patients from which plasma samples were used for investigating changes in soluble factors post fistuloplasty. “RC” denotes radio-cephalic, “BC” denotes brachiocephalic and “BVT” denotes basilic vein transposition fistula. “PIPP” denotes post intervention primary patency. Patients were followed up for a minimum of one year or until the end of PIPP or censoring.

Pre & Post Monocytes	Pre & Post Soluble Factors	Gender	Age (years)	Ethnicity	Type of Native Fistula	Fistula Age (years)	Previous Thrombosis	Previous Fistuloplasty	Reason for intervention	Fistula in use?	Medication	N. Days of follow up	Reason for the end of post intervention primary patency or for censoring (shaded grey)
✓	✓	F	56	Asian	BVT	4.80	N	Y	7	Y	N	1177	Kidney transplant
✓	✓	F	67	White	BC	4.51	N	Y	6	Y	A, S	84	Decision to Abandon
✓	✓	M	83	Black	BVT	0.21	N	N	2	N	A, S	114	Thrombosed
✓	✓	F	76	White	BC	5.86	N	Y	7	Y	W,S	624	Decision to Abandon
✓	✓	M	20	Asian	RC	1.76	N	N	2	Y	N	98	Thrombosed
✓	✓	M	60	Black	RC	0.08	N	N	2	N	P,T,M	280	Radiological Procedure
✓	✓	M	56	Black	BC	0.36	N	N	1	Y	N	109	Thrombosed
✓	✓	M	82	Black	BVT	4.82	N	Y	4	Y	P	562	Radiological Procedure
✓	✓	F	70	White	BC	1.19	N	N	2	Y	S	489	Thrombosed
✓	✓	M	68	Black	BC	0.54	N	N	2	N	A, S	921	Radiological Procedure
✓	✓	M	73	Black	BC	5.83	N	N	4	Y	A,W, S	158	Thrombosed
✓	✓	F	49	Black	BVT	1.04	N	Y	2	Y	N	501	Radiological Procedure
✓	✓	F	55	Black	BC	2.32	N	N	1	Y	A,	100	Radiological Procedure
✓	✓	F	37	White	RC	0.41	N	N	6	Y	N	140	Radiological Procedure
✓	✓	M	52	Black	RC	4.47	N	N	6	Y	A	823	End of Follow Up
✓	✓	F	33	White	BC	2.84	N	Y	4	Y	N	781	Radiological Procedure
✓	✓	M	53	White	BVT	0.59	N	N	2	Y	N	739	Death
✓	✓	F	38	Asian	BVT	1.10	N	N	3	Y	N	204	Radiological Procedure
✓	✓	M	88	White	BC	1.20	N	N	3	Y	S, P	291	Thrombosed
✓	×	F	63	White	BC	0.64	N	N	6	Y	A. S	294	Radiological Procedure
×	✓	M	62	White	RC	12.75	N	N	5	Y	S	230	Death
×	✓	F	59	Black	BC	0.24	N	N	6	Y	A	109	Thrombosed
×	✓	M	68	Black	RC	3.52	N	N	1	Y	N	1353	End of Follow Up
×	✓	M	70	White	BC	4.02	N	N	6	Y	N	52	Radiological Procedure
×	✓	M	60	Asian	BVT	0.31	N	N	2	Y	A	123	Surgical Intervention
×	✓	M	79	White	BC	1.85	Y	Y	2	Y	A, S	253	Decision to Abandon
×	✓	M	67	White	BC	1.40	N	N	3	Y	A, S	284	Surgical Intervention
×	✓	F	41	Black	BC	4.51	N	Y	3	Y	N	514	Kidney transplant
×	✓	F	62	Black	BC	2.31	N	N	7	N	N	371	Radiological Procedure
×	✓	M	62	Black	RC	0.627	N	N	2	Y	N	751	End of Follow Up

✕	✓	F	71	Black	BC	1.22	N	N	2	Y	S	71	Thrombosed
✕	✕	F	48	Black	BVT	2.72	N	N	4	Y	N	511	Radiological Procedure
✕	✕	M	48	Black	BVT	0.25	N	N	2	N	S	316	Radiological Procedure
✕	✕	F	70	Asian	BC	4.40	N	Y	7	Y	A, S	82	Decision to Abandon
✕	✕	F	45	Black	RC	1.81	N	Y	6	Y	A	252	Radiological Procedure
✕	✕	M	75	White	BC	1.44	N	N	7	N	N	84	Decision to Abandon
✕	✕	M	61	Black	BVT	0.98	N	N	2	Y	N	124	Thrombosed
✕	✕	F	49	Black	BVT	4.12	N	Y	6	Y	A	48	Kidney transplant
✕	✕	M	57	Black	RC	1.68	N	N	6	Y	N	180	Surgical Intervention
✕	✕	M	52	Black	RC	0.58	N	N	6	N	A,S	339	Thrombosed
✕	✕	F	71	Asian	BVT	0.53	N	N	6	Y	A	50	Radiological Procedure
✕	✕	M	31	White	BC	1.49	N	N	2	N	N	70	Kidney transplant
✕	✕	M	67	White	RC	3.41	N	N	6	Y	A, S	623	End of Follow Up
✕	✕	M	76	Asian	BVT	0.91	N	N	6	Y	A, S	215	Decision to Abandon
✕	✕	F	74	Black	BC	3.85	N	Y	6	Y	A, S	187	Decision to Abandon
✕	✕	M	61	Black	BC	1.48	N	N	3	Y	C	294	Radiological Procedure
✕	✕	F	49	White	BC	0.35	N	N	4	Y	N	366	Transplant
✕	✕	M	55	Black	BC	7.07	Y	Y	6	Y	N	138	Transplant
✕	✕	M	71	Black	BVT	2.54	N	N	2	Y	S	371	End of Follow Up
✕	✕	F	71	Asian	BVT	1.11	N	N	3	Y	NA	96	Decision to Abandon
✕	✕	M	71	Black	RC	0.98	N	N	7	Y	NA	460	Radiological Procedure
✕	✕	M	59	White	RC	0.95	N	N	6	Y	NA	199	Death
✕	✕	M	59	White	BC	0.60	N	N	2	Y	NA	389	End of Follow Up
✕	✕	F	48	White	RC	5.56	N	N	1	Y	NA	149	Surgical Intervention

Y= Yes, N=No. **Reason for intervention**; 1=inadequate dialysis, 2=decreased flow, 3=prolonged bleeding, 4=raised venous pressure, 5=decreased arterial pressure, 6=needling difficulties, 7=other. **Medication**; N=not on antiplatelets, statins, warfarin, prednisolone, or immunosuppressants. A=antiplatelet, S=statin, W=warfarin, P=prednisolone, T=tacrolimus, M=mycophenolate, NA=data not available.

Table S2. Soluble factors measured following a fistuloplasty (p and q values). Levels of 41 soluble mediators in paired patient plasma samples before and 1-2 days after their fistuloplasty. n = 30 paired samples. p values were calculated using a paired t test. False discoveries were account for using the method of Benjamini, Krieger and Yekutieli to give q values and are given in the table below. Data are shown in figure S1 below.

Factor	Mean Pre (pg/ml)	Mean Post (pg/ml)	Mean Change (Post - Pre)	p value	q value
Adiponectin	11537971.8	11021840.8	-516131.0	0.388	0.688
Angiogenin	969494.4	922324.2	-47170.2	0.399	0.688
Angiopoietin-1	11715.8	11334.0	-381.9	0.708	0.941
Angiopoietin-2	6782.5	6504.0	-278.6	0.282	0.688
BDNF	8229.6	9139.1	909.5	0.593	0.847
Calprotectin	1480776.1	1557052.3	76276.1	0.670	0.925
CD30	143.2	129.1	-14.1	0.076	0.670
CD40 Ligand	2685.2	2370.4	-314.8	0.173	0.688
CRP	4740132.8	4722230.6	-17902.2	0.847	0.941
CTACK	811.7	817.5	5.8	0.851	0.941
Eotaxin	697.1	707.3	10.2	0.338	0.688
Flt3 Ligand	90.9	96.2	5.3	0.278	0.688
HGF	516.3	514.0	-2.3	0.846	0.941
IL-16	706.5	703.1	-3.5	0.861	0.941
IL-17A	65.6	70.1	4.4	0.496	0.761
IL-18	408.9	397.6	-11.3	0.340	0.688
IL-1ra	1949.4	1794.5	-155.0	0.373	0.688
IL-2R α	1858.3	1855.3	-3.1	0.956	0.966
IL-6	1.2	2.1	0.9	**0.0003**	**0.012**
MCP-1	155.0	156.2	1.2	0.919	0.951
M-CSF	139.2	150.5	11.4	0.438	0.726
MIF	178945.3	152609.8	-26335.4	0.295	0.688
MIG	1813.8	1773.1	-40.8	0.212	0.688
MIP-3α	129.5	84.3	-45.3	0.463	0.737
MIP-3β	176.6	188.8	12.3	0.338	0.688
MMP1	2670.1	2542.3	-127.8	0.152	0.688
MMP8	5094.5	5663.3	568.8	0.312	0.688
MMP12	50.2	50.5	0.2	0.903	0.951
MMP13	1359.8	1380.8	21.0	0.291	0.688
MPO	110876.3	94430.5	-16445.8	0.040	0.548
PDGF-AA	909.5	821.0	-88.5	0.148	0.688
PDGF-BB	4331.1	3311.1	-1020.0	0.293	0.688
PF4	6316647.4	4150465.6	-2166181.9	0.194	0.688
RAGE	10245.1	10123.0	-122.1	0.783	0.941
RANTES	49453.8	62160.7	12706.9	0.350	0.688
S100A9	1946.8	1454.6	-492.3	0.758	0.941
S100B	881.3	862.6	-18.7	0.254	0.688
SCGF	10217.2	11275.2	1058.0	0.081	0.670
TfR	1476515.4	1646782.2	170266.8	0.555	0.820
TNF-α	15.6	16.6	1.0	**0.006**	**0.133**
VEGF	208.3	210.7	2.4	0.864	0.941

Figure S1. Soluble factors measured following a fistuloplasty (concentrations)
Levels of soluble mediators in paired patient plasma samples before and 1-2 days after their fistuloplasty. n = 30 paired samples. Each symbol pair represents a different patient. The p and q values for each factor are shown above in table S1. Data for IL-6 and TNFα are shown in the main paper.

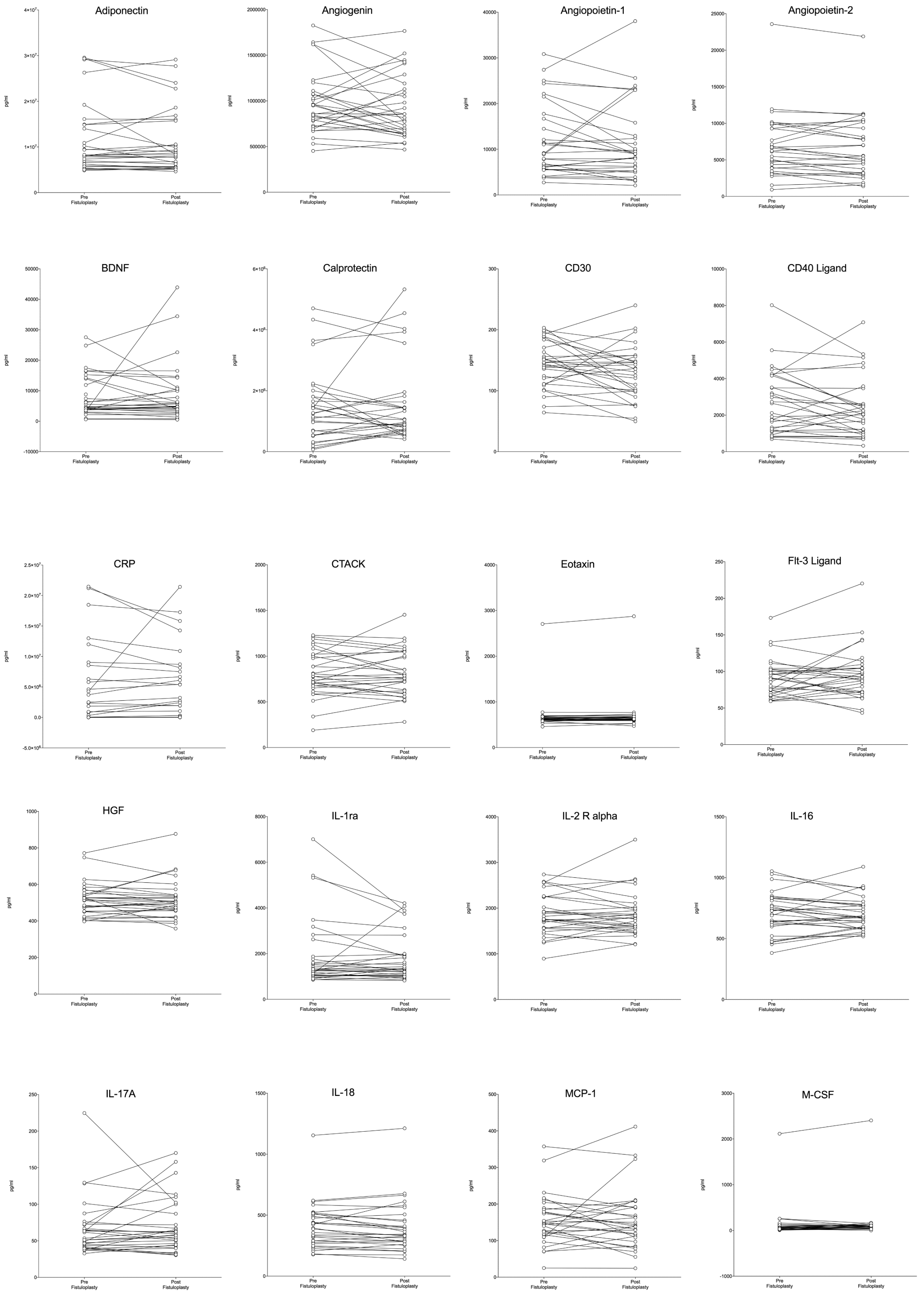

Figure S1. (continued)

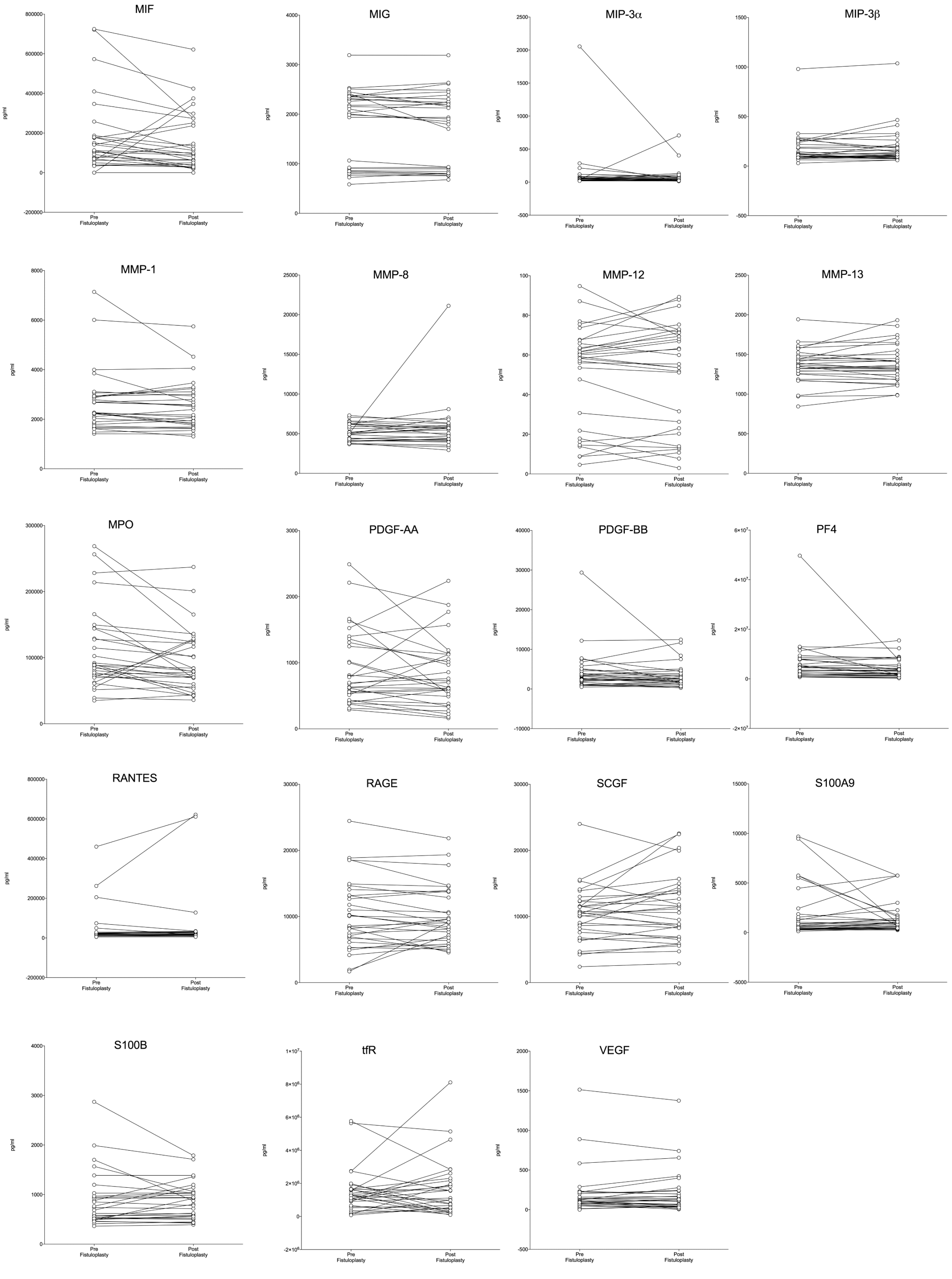

Table S3: Univariate cox regression analysis showing the association soluble factors with post intervention primary patency. Table showing the p values of the associations between the levels of each pre fistuloplasty protein, post fistuloplasty protein and change in protein level (calculated by subtracting post fistuloplasty with the pre fistuloplasty plasma protein levels) with either post-intervention primary patency or post-intervention cumulative patency outcome. (n = 54 patients for pre fistuloplasty plasma proteins, n = 30 post fistuloplasty and change in plasma proteins patient samples). Where p values were <0.05, false discoveries were calculated using the method of Benjamini, Krieger and Yekutieli to give q values.

Plasma Protein Time point	Pre Fistuloplasty		Post Fistuloplasty		Change (Post minus Pre)	
Protein	Significance	Hazard Ratio	Significance	Hazard Ratio	Significance	Hazard Ratio
Adiponectin	0.377	1.000	0.223	1.000	0.833	1.000
Angiogenin	0.124	1.000	0.671	1.000	0.852	1.000
Angiopoietin-1	0.944	1.000	0.451	1.000	0.632	1.000
Angiopoietin-2	0.744	1.000	0.917	1.000	0.934	1.000
BDNF	0.982	1.000	0.642	1.000	0.691	1.000
Calprotectin	0.257	1.000	0.496	1.000	0.625	1.000
CD30	0.858	1.000	0.430	1.004	0.464	1.003
CD40 Ligand	0.806	1.000	0.370	1.000	0.932	1.000
CRP	0.644	1.000	0.461	1.000	0.931	1.000
CTACK	0.442	1.000	0.983	1.000	0.294	1.001
Eotaxin	0.720	1.000	0.310	1.001	0.728	1.002
Flt3 Ligand	0.303	1.002	0.719	1.002	0.637	0.999
HGF	0.500	0.999	0.548	0.998	0.937	1.000
IL-16	0.570	1.000	0.654	0.999	0.451	1.002
IL-17A	0.898	1.000	0.715	0.998	0.885	0.999
IL-18	0.945	1.000	0.434	0.999	0.962	1.000
IL-1ra	0.462	1.000	0.439	1.000	0.758	1.000
IL-2R α	0.587	1.000	0.452	1.000	0.435	1.000
IL-6	0.431	0.871	0.216	0.838	0.160	0.769
MCP-1	0.330	1.002	0.567	1.001	0.940	1.000
M-CSF	**0.040** (0.828)	1.001	0.715	1.000	0.289	0.996
MIF	0.797	1.000	0.763	1.000	0.558	1.000
MIG	0.927	1.000	0.781	1.000	0.258	0.999
MIP-3α	0.531	1.000	0.239	0.997	0.162	0.999
MIP-3β	0.596	0.999	0.956	1.000	0.216	1.004
MMP1	0.899	1.000	0.962	1.000	0.637	1.000
MMP8	0.433	1.000	0.704	1.000	0.754	1.000
MMP12	0.775	1.002	0.650	1.004	0.649	1.010
MMP13	0.870	1.000	0.657	1.000	0.153	1.003
MPO	**0.003** (0.124)	1.000	0.325	1.000	0.111	1.000
PDGF-AA	0.976	1.000	0.407	1.000	0.894	1.000
PDGF-BB	0.909	1.000	0.237	1.000	0.195	1.000
PF4	0.164	1.000	0.657	1.000	0.134	1.000
RAGE	0.518	1.000	0.112	1.000	0.949	1.000
RANTES	0.224	1.000	0.319	1.000	0.410	1.000
S100A9	0.273	1.000	0.159	1.000	0.445	1.000
S100B	0.277	1.000	0.754	1.000	0.505	1.001
SCGF	0.502	1.000	0.761	1.000	0.988	1.000
TfR	0.804	1.000	0.351	1.000	0.334	1.000
TNF-α	0.198	1.025	0.338	1.022	0.894	0.981
VEGF	0.415	1.000	0.403	1.001	0.822	0.999
